# Midfoot and Forefoot Disorders in Adolescents and Adults with X-Linked Hypophosphatemia

**DOI:** 10.3390/jcm13226749

**Published:** 2024-11-09

**Authors:** Florian Wenzel-Schwarz, Celine C. Akta, Alexandra Stauffer, Adalbert Raimann, Roland Kocijan, Rudolf Ganger, Gabriel T. Mindler

**Affiliations:** 1 Department of Pediatric Orthopaedics and Foot Surgery, Orthopaedic Hospital Speising, Speisinger Strasse 109, 1130 Vienna, Austria; florian.wenzel-schwarz@oss.at (F.W.-S.);; 2 Vienna Bone and Growth Center, Währinger Gürtel 18–20, 1090 Vienna, Austria; 3 Department of Pediatrics and Adolescent Medicine, Division of Pediatric Pulmonology, Allergology and Endocrinology, Medical University of Vienna, Währinger Gürtel 18–20, 1090 Vienna, Austria; 4 Medical Faculty of Bone Diseases, Sigmund Freud University, Freudplatz 1, 1020 Vienna, Austria; 5 Ludwig Boltzmann Institute of Osteology at Hanusch Hospital of OEGK and AUVA, Trauma Centre Meidling, 1st Medical Department Hanusch Hospital, Heinrich-Collin-Straße 30, 1140 Vienna, Austria

**Keywords:** X-linked hypophosphatemia, foot, ankle, forefoot, hallux valgus, hypophosphatemia, deformity

## Abstract

**Objectives**: X-linked hypophosphatemia (XLH, OMIM 307800) is a rare genetic disorder that affects phosphate metabolism. While lower limb deformity represents a hallmark symptom of patients with XLH, the effect on the foot has not been investigated. This study aimed to characterise foot pathologies and assess related outcome scores in adolescents and adults with XLH. **Methods**: Patients aged ≥ 16 years with genetically verified XLH were included in this study. Physical examination was performed, and foot scores as well as foot X-rays were assessed. Radiographic analysis included the assessment of osteoarthritis, enthesopathies, and alignment abnormalities. **Results**: Twenty-six participants (51 limbs) with a mean age of 33.9 ± 15.4 years were eligible for the study. Patients with XLH presented with flatfoot deformity (reduced Meary angles > −4° in 84.3%), elevated first and fifth metatarsal angles > 30° (IM 1–5, 53.5%) and hallux valgus angles > 15° (36.0%). Moderate-to-severe joint space narrowing was observed in the talonavicular (49%) and cuneonavicular joints (41.2%). The American Orthopedic Foot and Ankle Society (AOFAS) score was associated with mildly reduced midfoot function. **Conclusions**: A high rate of bony deformity, joint degeneration, and decreased foot scores indicated the impact of forefoot and midfoot disorders in patients with XLH.

## 1. Introduction

X-linked hypophosphatemia (XLH, OMIM 307800) is a rare genetic disorder that affects phosphate metabolism, causing skeletal pain and impaired mobility. The disease is caused by a loss-of-function mutation in the *PHEX* gene (phosphate-regulating gene with homology to endopeptidases on the X chromosome), which increases fibroblast growth factor 23 (FGF-23) production, leading to renal phosphate wasting and reduced activation of 1,25 vitamin D [1]. In the UK, this condition affects approximately 1.5 children and 1.6 adults per 100,000 individuals [1]. The disease also manifests with bone or joint pain, gait abnormalities, and other functional impairments, which significantly impact patients’ quality of life [1,2,3,4].

XLH is commonly diagnosed in early childhood and is associated with short stature, rachitic growing plates, musculoskeletal pain, complex skeletal deformities, lower limb disabilities, premature osteoarthritis (OA), and enthesopathies [1,2,3,4]. Owing to these complex skeletal deformities, quality of life (QoL) is reduced in patients with XLH [2,3,4], with a majority of patients reporting joint problems as a burden of disease [3,4].

A recently published study reported that approximately one-third of adults with XLH report ankle bone pain, and half report ankle joint pain. Joint pain of the foot and toes affects 40.5% and 20.3% of adults with XLH, respectively [3]. Premature osteoarthritis (OA) development in the ankle is a known characteristic of XLH [2]. Research underscores the necessity for comprehensive treatment strategies, encompassing both nonsurgical and surgical approaches, to address foot and ankle disorders in XLH patients [5]. Moreover, investigations have systematically quantified lower limb deformities and gait abnormalities across various age groups affected by XLH [6,7,8].

However, no details on midfoot and forefoot foot deformities or on specific patient-reported scores have been elucidated to date. The origin of the reported foot pain in patients with XLH [3] remains unclear, necessitating further foot-specific examinations in this population. Interpreting foot-related symptoms in patients with XLH is challenging due to the multitude of potential disease-specific changes, such as deformities, osteoarthritis, and enthesopathies. This complexity underscores the need for a systematic analysis of the feet in patients with XLH.

After having previously reported on ankle problems in XLH patients [2], the aim of this study was to evaluate and specify midfoot and forefoot deformities, the presence of OA, enthesopathies, QoL, and patient-related outcomes measures (PROMs) in adolescents and adults with XLH. The study’s intention was to highlight the need for special consideration and treatment of this disease-specific issue. Additionally, the findings of this study may assist surgeons in developing timely, tailored treatment options for this particular foot impairment, as there is currently no established treatment algorithm in the literature.

## 2. Material and Methods

A single-centre, cross-sectional analysis of adolescent and adult patients with XLH, with and without previous surgical intervention, was performed at the Orthopaedic Hospital Speising, Vienna, Austria. Patients with confirmed XLH and ≥16 years of age were invited to participate in this study to evaluate early OA and osteophyte development. Patients were actively recruited via the Austrian XLH patient organisation to participate in this study with a radiographic evaluation, clinical examination, and foot/QoL scores. Additionally, radiographs were retrospectively included from our disease-specific database. Written consent was obtained from all participating patients, with parental consent provided for those under 18 years. The exclusion criteria were other forms of hypophosphatemia, pregnancy, non-weight-bearing X-rays, and recent lower limb surgery within 3 months. The study was approved by a local ethics committee (EK37/2020, amendment approved February 2021).

During a period of 5 months (March 2021 to July 2021), data from 28 patients with XLH were available for this study. Two adults were excluded due to pregnancy. Radiographic data (both from invited patients and retrospective data base analysis) from a total of 26 adolescents and adults with XLH (51 legs) and clinical data (invited patients only) from a total of 37 limbs were included in this study. One foot was excluded from the analysis because of recent surgical intervention at the knee. The study cohort overlaps with the study population of Akta et al. [2].

The clinical examination of all invited patients was conducted by a board-certified foot and ankle surgeon (FWS). The clinical assessment included a questionnaire and physical evaluation from the AOFAS score, as well as testing and scoring multiple pressure points as ‘positive’ or ‘negative’, including the flexor hallucis longus tendon, Achilles tendon insertion, plantar fascia, sinus tarsi, talonavicular joint, navicular–cuneiform joint, and the Lisfranc joint line. Hindfoot alignment was evaluated while standing and categorised as varus, neutral, or valgus. The peroneal tendons were assessed for pressure pain, thickening, subluxation/luxation, and insufficiency. Specific ankle and hindfoot parameters were also assessed, including passive and active range of motion measured with a handheld goniometer, a Silverskjöld test, a dorsiflexion lunge test, and ankle stability tests. These ankle-specific results have been reported by Akta et al. [2]. Prior foot surgeries and current medical therapies were recorded.

Radiographs of the ankle and foot were taken in the anteroposterior/dorsoplantar views while the patient was standing with a centralised beam on the ankle joint. On the dorsoplantar radiographs, the talocalcaneal angle (TCA), talo-metatarsal 1-angle (TMT-1), 1st to 2nd intermetatarsal angle (1–2 IMA), hallux valgus angle (HVA) and intermetatarsal angle between the 1st and 5th metatarsals (IM 1–5) were measured. “Hallux valgus” was defined as an HV angle > 15° [9]; on lateral radiographs, the Meary angle and the calcaneal inclination angle were measured (Supplemental Figure S1). The lateral distal tibial angle (LDTA) was measured on anteroposterior ankle X-rays. The radiographic measurements were then compared with published reference ranges [9,10,11,12]. The reference ranges are detailed in Table 1.

Radiographic analysis and scoring of the foot and ankle were performed by a specialised foot surgeon (FWS) using Siemens Syngo imaging software (version number: VB36E Siemens Healthineers AG, Siemensstr. 3, 91301 Forchheim, Germany). OA was visually analysed according to the modified Kellgren–Lawrence osteoarthritis score [13]. Six joints were included in the grading system: the talonavicular (TN), cuneonavicular (CN), calcaneocuboid (CC), metatarsocuneiform (TMT1), first metatarsophalangeal (MTP1) and Lisfranc joints.

OA was graded (Kellgren–Lawrence score) as “no radiographic findings of osteoarthritis”, “minute osteophytes of doubtful clinical significance”, “definite osteophytes with mild joint space narrowing”, “definite osteophytes with moderate joint space narrowing”, and “definite osteophytes with severe joint space narrowing”. The results for OA ranged from “definite osteophytes with mild JSN (joint space narrowing)” to “definite osteophytes with severe JSN” if not stated otherwise.

All X-rays were analysed for signs of pseudofractures.

The American Orthopedic Foot and Ankle Society (AOFAS) score (midfoot section and 1st forefoot ray, forefoot rays 2–5) and the foot function index (FFI) were used to assess foot function and QoL [14]. The AOFAS midfoot and forefoot section included questions addressing pain, function, shoe wear, walking distance, issues with walking surfaces, gait abnormalities, foot alignment, and joint motion. The FFI, along with its subscales—pain, activity, and disability—was collected from each patient. It included questions addressing foot pain, specifically inquiring about worst pain, pain during activities, and time of day when pain occurs. It also covered foot function, with questions on functional abilities during walking, stair climbing (up and down), tiptoeing, sports, and shoe wear.

### Statistics

Statistical analysis was performed using SPSS (IBM SPSS Statistics, version 27). The standard distribution was assessed using the Kolmogorov–Smirnov test, and descriptive statistics were calculated for both the radiological and the clinical results. One-way analysis of variance (ANOVA) was employed to determine the relationship between clinical changes in radiological findings and functional and QoL scores (AOFAS, FFI) across variables with two or more groups, with a significance level set at α < 0.05. Levene’s test, normality checks, and homogeneity tests were performed, and the assumptions were met. For parameters with normal and non-normal distributions, Pearson’s or Spearman’s correlations were calculated, respectively. Data with a normal distribution were assessed via the Shapiro–Wilk test.

## 3. Results

### 3.1. Study Population and Demographics

A total of 26 adolescents and adults with XLH (51 legs, 20 females, six males, mean age: 33.9 ± 15.4 years, 16–72 years), of whom 19 were available for physical examination, were included in this study. Our study cohort had a decreased standing height (mean: 155.3 ± 8.0 cm, 138–167 cm) compared with the Austrian general population [15] and a mean weight of 69.5 ± 12.2 kg (52–97 kg); the mean BMI was 29.2 ± 6.8 kg/m^2^ (18.4–42.2), which is considered preobese [16]. Phosphate and vitamin D3 therapy were actively taken by 13 XLH patients. No patient had burosumab therapy at the time of examination.

Patients reported having undergone bony lower limb surgery an average of 5.6 times per person (±4.9; range: 0–17). Only one patient with XLH reported having previously undergone surgery on the foot (endoscopic ablation of the dorsal calcaneal spur). The summary characteristics are listed in Table 2.

### 3.2. Radiographic Results

#### 3.2.1. Foot Deformity Analysis

Patients with XLH presented with flatfoot deformity defined by a reduced Meary angle > −4° in 84.3% of cases (mean: −8.4° ± 10.3°, range: −39.8 to +11.0°). Hallux valgus deformity, defined as hallux valgus angle > 15°, was detected in 36% (mean HV angle 13.3° ± 7.7°, range: 1–29.5°). The intermetatarsal angle between metatarsus 1 and 5 was elevated (>30°) in 30% of patients (mean: 24° ± 4°, range: 15.5–31.7°), indicating splayfoot deformity. The mean anteroposterior talocalcaneal angle was within the upper normal range (mean: 24.9° ± 5.3°; 15–37°). The mean calcaneal inclination angle was within normal range (mean: 13.9° ± 4.8°, range 1.2–24.5°).

The radiographic measurements and normal values are summarised in Table 1.

#### 3.2.2. Osteoarthritis, Enthesopathies, Pseudofractures

**Figure 1:**
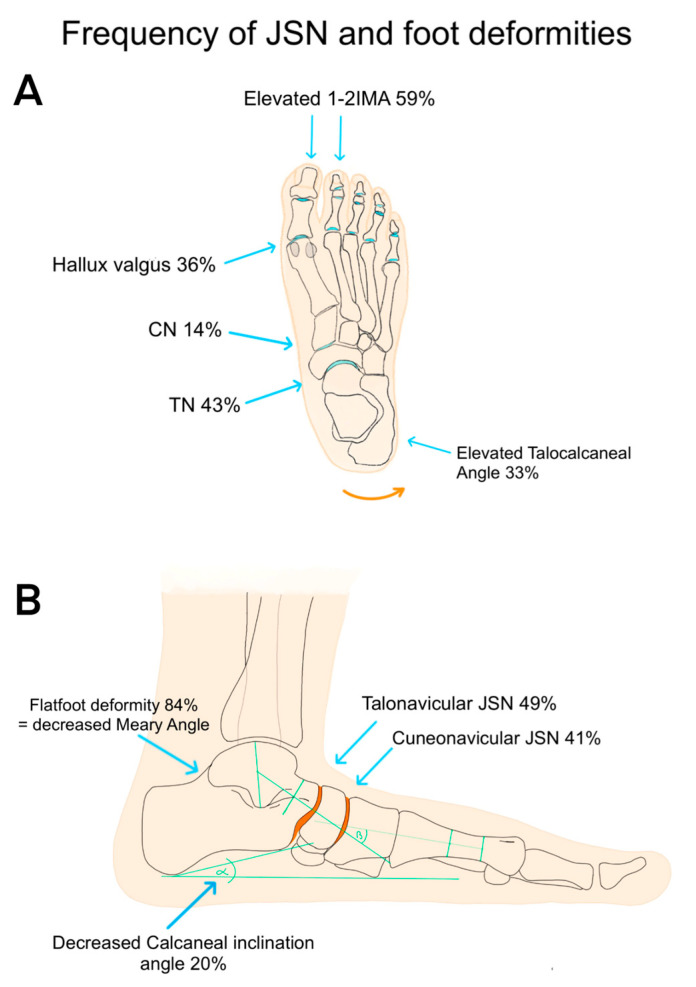
Frequency of deformity and JSN of the foot: the orange surface indicates predilection sites for OA. (**A**): Dorsoplantar view; (**B**): lateral view (source: Celine C. Akta).

According to the lateral radiographs, the talonavicular (49.0%) and cuneonavicular joints (41.2%) were the most prominent sites of OA, presenting definite osteophytes with mild JSN. The TMT1 (13.7%) and MTP1 joints (7.8%) were less affected by osteophytes and JSN (Figure 1).

**Figure 2:**
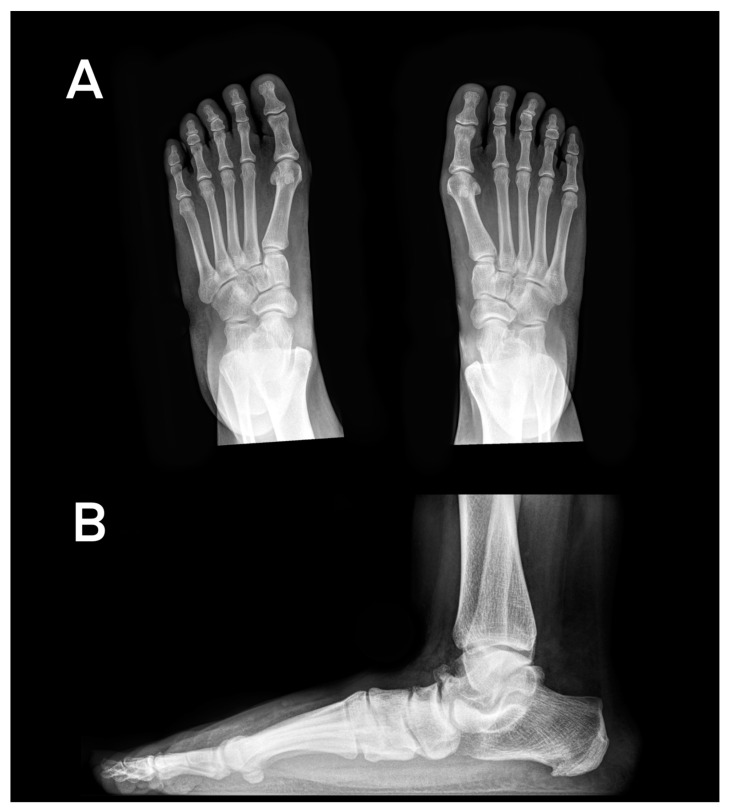
(**A**): Weight-bearing, dorsoplantar radiographs of the feet presenting with hallux valgus (HVA > 15°), elevated TCA, and mild JSN of the talonavicular joint. (**B**): Lateral radiograph of the left foot and ankle presenting with moderate JSN of the TN joint, mild JSN of the CN joint, and a decreased Meary angle and calcaneal inclination angle.

**Figure 3:**
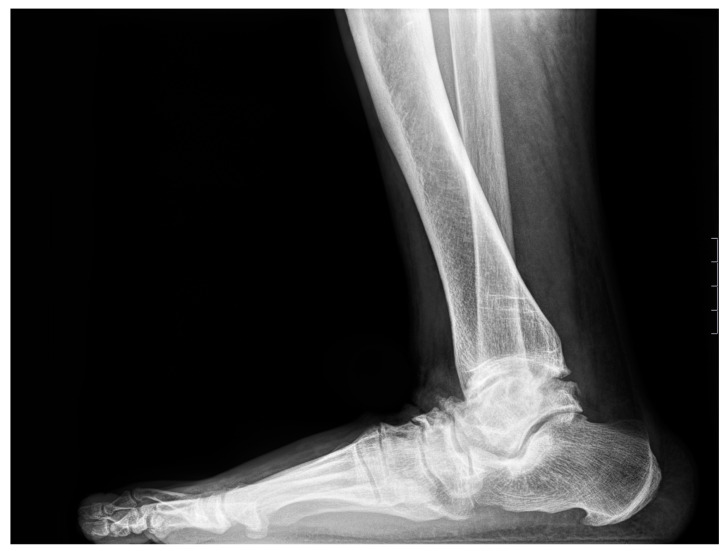
Lateral radiograph of the foot: 50-year-old female patient presenting with enthesopathies of the talus and navicular bones and severe JSN of the TN and NC joints.

Dorsoplantar radiographs revealed TN OA in twenty-two limbs (43.1%); nineteen limbs (37.2%) presented with OA of the CN joint, and seven limbs (13.8%) presented with CC joint. Seven feet (13.8%) presented with TMT1, four feet (7.8%) were affected with MTP1, and five feet (9.8%) presented with Lisfranc OA. The most prominent sites for OA and foot deformities are displayed in Figure 1, Figure 2 and Figure 3.

No pseudofractures of the metatarsal bones have been detected.

### 3.3. Physical Examination

Varus and valgus malalignment of the hindfoot were detected in eight out of fifty-one limbs (21.6%), respectively, whereas twenty-one limbs had a neutral alignment (56.8%). Pressure pain in the tibialis posterior tendon was reported in four limbs (10.8%), and pressure pain in the flexor hallucis longus tendon was reported in three limbs (8.1%). Six out of thirty-seven feet (16.2%) presented pressure pain in the plantar fascia, and four feet had pressure pain in the loco typico (66.6%). Four patients experienced pressure pain in the sinus tarsi (17.1%), the talonavicular joint space (25.7%) and the Lisfranc joint space (20.0%). Only two limbs tested positive for navicular and medial cuneiform pressure pain.

#### 3.3.1. Correlations

There was no significant correlation between a positive Silverskjöd test score and pressure pain or calcifications in the Achilles tendons (*p* > 0.05). There was no significant correlation between the distal lateral tibial angle (ankle deformity) and the ap talocalcaneal angle or Meary angle. Distal tibial varus deformity (radiologic assessment) was not associated with increased varus deformity of the heel or increased medial arch height (in the clinical examination).

#### 3.3.2. Foot Function Scores

Foot function scores were available for 37 feet (Table 2).

AOFAS (high scores indicate no pathologies): midfoot, 89.8 ± 14.2% (range: 43–100%); metatarsophalangeal-interphalangeal for the hallux, 95.2 ± 7.7% (range: 73–100%); and metatarsophalangeal-interphalangeal for the lesser toes, 96.7 ± 6.4% (range: 75–100%).

The FFIs (high scores indicate higher impairment) were as follows: symptoms, 78.9 ± 20.4% (range: 31.9–100%); function, 74.8 ± 26.1% (range: 13.3–100%).

## 4. Discussion

The main aim of this study was to identify and systematically evaluate foot-related symptoms originating from midfoot and forefoot disorders in adolescents and adults with XLH. Foot-related symptoms, deformities such as flat feet, the presence of OA, enthesopathies, and QoL were documented.

Our findings highlight the significant prevalence of flatfoot deformity and hallux valgus, alongside a high occurrence of moderate-to-severe joint space narrowing in the talonavicular and cuneonavicular joints. The functional limitations were more pronounced in the midfoot than in the forefoot, as indicated by the AOFAS score. Moreover, the FFI indicated reduced QoL regarding foot function and symptoms.

Recent studies [2,3,4] have explored lower limb pathologies and their impact on QoL in people with XLH. Akta et al. [2] investigated ankle function in adolescents and adults with XLH and reported that subtalar ankle OA reduced QoL on the basis of patient-reported outcome scores and that clinical ankle restrictions resulted in lower gait quality and ankle power.

As a follow-up, this study elucidates disorders of the midfoot and forefoot in people with XLH. Additionally, pathologies in the forefoot, such as hallux valgus and the presence of OA in the metatarsophalangeal joints, were examined. Overall, foot deformities (flatfoot, splayfoot, etc.) were evaluated via various radiographic angles. The physical examination included identifying painful pressure points, such as the plantar fascia, the insertion of the Achilles tendon, and the tibialis posterior and flexor hallucis longus tendons.

An acquired flatfoot deformity, also known as planovalgus deformity, is characterised by medial rotation and plantar flexion of the talus, the eversion of the calcaneus, and a collapsed medial arch. This phenomenon is primarily due to dysfunction or weakening of the posterior tibial tendon, along with weakening of other talocalcaneal and tarsal ligaments, and it is further associated with hindfoot valgus. This condition can destabilise the arch, leading to increased midfoot abduction and forefoot supination [11].

The prevalence of acquired flatfoot deformity in adults in the general population ranges between 5% and 37%, depending on the literature, with rates exceeding 10% in adults over the age of 65 [17]. In comparison, our patient cohort is considerably younger, which may be attributed to the disease’s direct impact on the cellular structure of bones, tendons, and soft tissues.

In a recent study with an overlapping cohort, we reported distal tibial varus deformity in 30% of the patients and valgus deformity in 22% of the patients [2]. Clinical examination of our current cohort revealed heel varus in 17.5% of the patients, with most feet exhibiting a tendency towards flatfoot deformity. Lateral distal tibial deformity (LDTA) did not correlate with foot deformity parameters (ap talocalcaneal angle, Meary angle), suggesting the independence of distal tibial varus or valgus deformity in our cohort of XLH patients. The propensity for flatfoot deformity in individuals with XLH is highly relevant in the context of planning lower limb reconstruction procedures. Orthopaedic surgeons are faced with the question of whether correcting distal tibial deformity (varus correction) exacerbates flatfoot deformity in individuals with XLH. This possibility makes it challenging for surgeons to determine the optimal timing for the correction of distal tibial deformity to prevent subtalar compensation. Additionally, the impact of tibial malrotation on foot and ankle function must be taken into consideration [2].

More than two-thirds of our study population presented a decreased Meary angle, indicating flatfoot deformity. We hypothesise that the progressive lowering of the medial arch is due to degeneration, mainly of the TN and CN joints. However, a decreased Meary angle did not correlate with the position of the heel, potentially because patients with XLH present with degenerative changes and early signs of OA accompanied by recurrent inflammation resulting in premature joint stiffness [2,18], which may cause a malfunction in the interaction between the forefoot and the hindfoot.

While tibialis posterior tendon insufficiency or symptoms are common in patients with flatfeet, only one-tenth of the patients in this cohort presented related symptoms on clinical examination, and even fewer complained about pain concerning the flexor hallucis longus tendon. This finding could be attributed to the relatively young mean age of our cohort. In contrast, more patients reported pressure pain in the Chopart talonavicular joint space and the Lisfranc joint space of the sinus tarsi. A plausible explanation for this phenomenon seems to be that OA and osteophytes were predominantly observed in the TN joint and the CN joint.

The midfoot osteoarthritis rate in patients with XLH appears astonishingly high compared to the general population, where the prevalence of midfoot osteoarthritis is reported at 3.5% among individuals over the age of 20 [19].

As expected, subjective symptoms, degeneration and signs of OA decreased in the evaluation of the forefoot from proximal to distal in our cohort. Compared with the other joints, the TMT1 and MTP1 joints were less affected by osteophytes and JSN.

Overall, multiple factors, such as a bony anatomy, joint positioning, and ligament and tendon integrity, are essential for proper foot alignment and function. Therefore, assessing foot alignment and function is particularly relevant in conditions such as XLH, demonstrating symptoms such as enthesopathy, early osteoarthritis, osteomalacia, deformity, and chronic pain.

We agree with other authors that the definition and quantification of enthesopathies is challenging [2,20]. Under normal circumstances, the fibrocartilaginous enthesis develops postnatally and is found at insertions into epiphyseal or apophyseal bones with greater mechanical loads. It resists deformation in response to tensile forces and withstands failure in response to mechanical stress during unloading conditions, thus transferring substantial muscular forces to the skeleton. In cases of osteomalacia, the loss of mineralised fibrochondrocyte tethering results in increased elongation of the tendon rather than progressive stabilisation at the insertion site. Furthermore, softer subchondral bone causes excessive strain on the fibrocartilaginous transition tissue and creates a mechanical stimulus for cellular adaptation, resulting in the expansion of mineralising fibrochondrocytes. These cells contribute to the development of bone spurs or enthesophytes, thus accounting for symptoms in the clinical profile of patients with XLH [21].

We previously reported a high rate of calcification around the insertion of the Achilles tendon, dorsal calcaneal spurs, and plantar calcaneal spurs in a population with a mean age of 32.9 years [2]. Additionally, osteophytes were present in the talonavicular and cuneonavicular joints in the present study of an overlapping population. Although enthesopathies and pain are well documented as part of the clinical profile of adults with XLH [3,4,20], the differentiation between tibialis posterior tendinopathy due to insufficiency in cases of flatfoot deformity and enthesopathy caused by underlying metabolic diseases remains challenging in clinical practice when determining the cause of foot pain in XLH.

Pseudofractures have been primarily reported in the lower limbs of patients with XLH, with an unclear pathomechanism. In a previous study, we reported a pseudofracture rate of 30% in an overlapping cohort of adolescents and adults with XLH [7]; however, we did not find signs of pseudofractures in the metatarsal bones in this study. Bony deformities have been discussed as potential contributors to pseudofracture development. Nevertheless, in our previous study, pseudofracture occurrence did not correlate with measurements of bone deformity [7]. Despite a high rate and severity of foot deformity, which alters forces and plantar pressure distribution, this does not appear to provoke pseudofracture development of the metatarsal bones.

With respect to foot function and QoL, the FAOS score revealed a general reduction in foot and ankle function among individuals with XLH [2]. However, the AOFAS score, which differentiates anatomical areas (hindfoot, midfoot, forefoot), demonstrated good function in the forefoot but decreased function in the midfoot and hindfoot among adolescents and adults with XLH (as previously reported in this overlapping cohort [2]).

However, the outcomes of this study present a controversial analysis: despite a high rate of deformity and osteoarthritis detected, the foot-specific scores were not particularly poor, and only one patient in our cohort had prior corrective foot surgery. Reasons for this discrepancy could include the possibility that patients with XLH are accustomed to chronic pain or that patients who had undergone prior surgeries had more significant issues, leading them to overlook foot pain. Additionally, it may relate to orthopaedic surgeons being somewhat hesitant due to a lack of knowledge about the disease or insufficient data on surgical outcomes for foot surgeries in XLH.

There is a lack of information on orthopaedic foot and ankle treatment in patients with XLH. While deformity, OA, and symptoms are present at a young age (Skrinar et al. [3] reported foot joint pain in 40.5% and toe pain in 20.3% of adults), to the best of our knowledge, no study has reported surgical or conservative treatment of forefoot and midfoot procedures in XLH. Specifically, no data on the rate of hallux valgus correction are available. The high rate of foot disorders observed in the present study does not necessarily correspond to a need for more surgical treatment. The indications for surgical procedures must be considered cautiously, as chronic pain is a main symptom of XLH and affects not only joints but also bone and muscle [1]. With respect to conservative treatment options, insoles are highly recommended to prevent further lowering of the medial arch. Physical therapy, along with gait training starting at a young age, may also help prevent further deformities and aid in maintaining good foot function in the future. For the conservative management of OA, injection therapy with hyaluronic acid or autologous blood injections might be a treatment option, although data regarding conservative treatment of XLH patients are missing.

Systematic follow-up studies on the treatment results for foot and ankle treatments in XLH are needed to define the best treatment algorithm for this complex disease.

Our data showed that patients with XLH presented with a high rate of disorders of the foot from an early age. Although foot function is crucial in everyday life, compared with deformities of the lower limb, especially the hip or the knee, it has not received sufficient scientific attention despite being highly clinically relevant. Further studies, conducted in close collaboration with patients and focusing on their needs, are necessary to fully understand this rare metabolic disease.

## 5. Limitations

This study had several limitations. While recent studies have analysed lower limb deformity in nonsurgically treated patients with XLH [6,22], our study cohort was heterogeneous with respect to prior surgical treatments.

Although maltorsion is an essential part of lower limb deformity in XLH [7], torsional MRI or CT scans were not available in this study. Furthermore, MRI of the foot provides important information on cartilage and ligament status, whereas pedobarography provides more details on pressure distribution during weight bearing.

## 6. Conclusions

A high rate of bony deformity and joint degeneration as well as decreased foot-specific scores indicate the impact of forefoot and midfoot disorders on the QoL and daily activity of patients with XLH. Clinical and radiographic examinations revealed several underlying causes of foot pain in patients with XLH.

Surgical deformity correction of the distal tibia and foot in patients with XLH requires a comprehensive deformity analysis, including clinical assessment of foot deformities. This is necessary because, unlike in the general population, distal tibial deformity, heel alignment, medial arch alignment, and foot symptoms do not always correlate in patients with XLH. To plan surgical procedures for the foot and ankle, XLH-specific pathologies must be considered. Clinicians should pay close attention to foot complaints in XLH, considering factors such as foot architecture, early osteoarthritis, and the presence of enthesiopathies.

## Figures and Tables

**Table 1:** Comparison of deformity data between XLH patients and the reference normal range.

	N (Limbs)	Mean	SD	Min	Max	Normal Reference Range
Meary Angle	51	−8.4	10.3	−39.8	11.0	0 ± 4 [12]
Calcaneal inclination angle	51	13.9	4.8	1.2	24.5	10–20 [12]
TCA (dp)	51	24.9	5.3	15.0	37.0	15–27 [12]
TMT-1	51	8.4	5.3	0.4	19.6	0–3 [10]
1–2 IMA	51	10.4	2.7	3.2	16	6–10 [12]
1–5 IMA	51	24.0	4.0	15.5	31.7	<30 [12]
HVA	51	13.3	7.7	1	29.5	<15 [9]

**Table 2:** Characteristics of the included patients with XLH.

	Mean	SD	Min	Max
Age (years)	33.9	15.4	16	72
Weight (kg)	69.5	12.2	52	97
Height (cm)	155.3	8	138	167
BMI (kg/m^2^)	29.2	6.8	19.1	42
AOFAS				
MTP-IP ** hallux	95.2	7.7	73	100
MTP-IP ** lesser toes	96.7	6.4	75	100
FFI * (%)	76.9	23.3	13.3	100
FAOS *** (%)	76.9	25.3	11.9	100

* Higher scores indicating higher impairment, ** metatarsophalangeal-interphalangeal, *** data from previous study by Akta et al. with an overlapping cohort [2].

## Data Availability

Dataset available on request from the authors.

## Supplementary Materials

The following supporting information can be downloaded at: https://www.mdpi.com/article/10.3390/jcm13226749/s1, Figure S1: Graphical illustration of measured angles on dorsoplantar (A) and lateral (B) radiographs. On the dorsoplantar radiographs (A) the talocalcaneal angle (a), the tarso-metatarsal 1 angle (b), the 1st to 2nd intermetatarsal angle (c), hallux valgus angle (d) and intermetatarsal angle between the 1st and 5th metatarsals (IM 1–5) were measured. On lateral radiographs (B), the Meary angle (f) and the calcaneal inclination angle (g) were measured.
