# Supplementary material for: Midfoot and Forefoot Disorders in Adolescents and Adults with X-Linked Hypophosphatemia
Source: J Clin Med. 2024 Nov 9;13(22):6749. doi: 10.3390/jcm13226749 (PMC11594587; doi:10.3390/jcm13226749)
Supplement: Supplementary file 1 [file jcm-13-06749-s001.zip › jcm-3244211-supplementary.pdf]

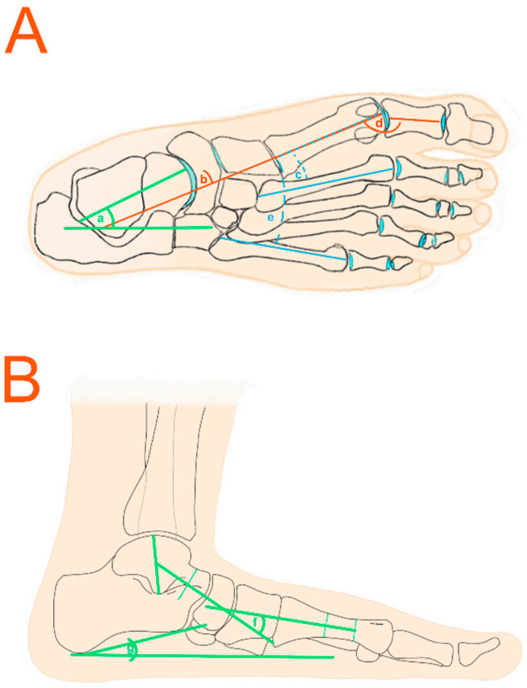

Supplemental figure S1. Graphical illustration of measured angles on dorsoplantar (A) and lateral (B) radiographs. On the dorsoplantar radiographs (A) the talocalcaneal angle (a), the tarso-metatarsal 1 anlge (b), the1st to 2nd intermetatarsal angle (c), hallux valgus angle (d) and intermetatarsal angle between the 1st and 5th metatarsals (IM 1–5) were measured. On lateral radiographs (B), the Meary angle (f) and the calcaneal inclination angle (g) were measured.
